# A PCR, qPCR, and LAMP Toolkit for the Detection of the Wheat Blast Pathogen in Seeds

**DOI:** 10.3390/plants9020277

**Published:** 2020-02-21

**Authors:** Maud Thierry, Axel Chatet, Elisabeth Fournier, Didier Tharreau, Renaud Ioos

**Affiliations:** 1 ANSES Plant Health Laboratory, Mycology Unit, Domaine de Pixérécourt, Bâtiment E, F-54220 Malzeville, France; maud.thierry971@gmail.com (M.T.); axel.chatet@anses.fr (A.C.); 2 UMR BGPI, Montpellier University, INRAE, CIRAD, Montpellier SupAgro, 34398 Montpellier, France; elisabeth.fournier@inrae.fr (E.F.); didier.tharreau@cirad.fr (D.T.); 3 CIRAD, UMR BGPI, F-34398 Montpellier, France

**Keywords:** seed testing, diagnostic, *Pyricularia oryzae*

## Abstract

Wheat blast is a devastating disease caused by the pathogenic fungus *Pyricularia oryzae.* Wheat blast first emerged in South America before more recently reaching Bangladesh. Even though the pathogen can spread locally by air-dispersed spores, long-distance spread is likely to occur via infected wheat seed or grain. Wheat blast epidemics are caused by a genetic lineage of the fungus, called the *Triticum* lineage, only differing from the other *P. oryzae* lineages by less than 1% genetic divergence. In order to prevent further spread of this pathogen to other wheat-growing areas in the world, sensitive and specific detection tools are needed to test for contamination of traded seed lots by the *P. oryzae* *Triticum* lineage. In this study, we adopted a comparative genomics approach to identify new loci specific to the *P. oryzae* *Triticum* lineage and used them to design a set of new markers that can be used in conventional polymerase chain reaction (PCR), real-time PCR, or loop-mediated isothermal amplification (LAMP) for the detection of the pathogen, with improved inclusivity and specificity compared to currently available tests. A preliminary biological enrichment step of the seeds was shown to improve the sensitivity of the tests, which enabled the detection of the target at an infection rate as low as 0.25%. Combined with others, this new toolkit may be particularly beneficial in preventing the trade of contaminated seeds and in limiting the spread of the disease.

## 1. Introduction

Wheat blast is an emerging disease threatening global wheat production and thereby global food safety. The major agricultural impact of the disease and its rapid propagation require a robust tool for reliable and timely identification of the pathogen in order to prevent its spread and avoid further exchange of contaminated biological material at a global scale.

The pathogen responsible for wheat blast (*Pyricularia oryzae Triticum* lineage, synonym *Magnaporthe oryzae*) is capable of infecting all aerial parts of the wheat plant, but spike infection is the most common symptom observed in the field [1,2,3]. Symptoms of wheat blast include necrotic lesions on leaves, stems, grains, and partial or total bleaching of the spikes leading to sterility or empty grains. Wheat blast disease reduces grain yield and grain quality [2,4]. During major epidemics, it has caused up to 100% yield loss [5]. The disease could potentially have a significant economic impact since cereals account for about 40% of the world’s agricultural yield, with wheat coming second in 2017 after maize and before rice, and reaching more than 750 M t produced and 500 M t consumed annually by humans [6].

This devastating disease first emerged in 1985 in the state of Paraná in Brazil [7]. The disease then spread to the neighboring federal states of São Paulo and Mato Grosso do Sul in 1986, and Rio Grande do Sul in 1987, causing a decrease of 95% in the wheat crop yield of the Cerrado ecoregion [8,9,10,11]. The pathogen subsequently spread to eastern Bolivia in 1996, eastern Paraguay in 2002, and northern Argentina in 2007 [5]. It then broke out in Bangladesh in 2016 [12,13].

*P. oryzae* spores are mainly dispersed on short distance by the wind [14]. However, the transport of contaminated seed or grains facilitates the spread of the fungus over long distances [15]. Comparative genomic studies demonstrated that *P. oryzae* isolates collected from wheat in different parts of Bangladesh and isolates causing epidemics in Brazil were strongly genetically related [12,13]. The transport of grains contaminated with *P. oryzae*, harvested in the wheat blast epidemic zone in Brazil, likely caused the recent emergence of this pathogen in Bangladesh in 2016 [12].

*P. oryzae* is responsible for blast disease on numerous Poaceae species [16,17]. Phylogenetic analyses of 81 genomes of *P. oryzae* isolates sampled from 12 different genera of Poaceae revealed multiple divergent lineages within *P. oryzae*, each of which was mostly associated with one specific host plant genus [18]. The existence of these host-specific lineages revealed incipient speciation, following host jump or host range expansion of the pathogen. However, the genetic divergence (number of differences per kilobase) observed between host-specific lineages was less than 1% on the entire genome—by comparison, the genetic divergence between different species of *Pyricularia* (*P. grisea, P. oryzae,* and *P. pennisetigena*) is greater than 10%—and gene flow was detected between host-specific lineages [18]. *P. oryzae* therefore represents a single species grouping different host-specific lineages.

The majority of isolates sampled from infected wheat cluster within one of these host-specific lineages, the *Triticum* lineage. However, *P. oryzae* isolates are sometimes able to opportunistically infect a host plant different from their original host, but this causes far fewer symptoms. Several studies have recorded opportunistic infection of wheat caused by isolates belonging to the *Lolium* lineage of *P. oryzae*, which is genetically the closest host-specific lineage to the *Triticum* lineage. These isolates, however, were weakly aggressive on wheat during artificial inoculation tests and do not appear to be capable of causing major epidemics on this host [3,19]. The *Triticum* lineage of *P. oryzae* is therefore considered responsible for wheat blast epidemics.

Accurate and rapid methods for detecting wheat blast isolates are required to limit or prevent the spread of the pathogen in disease-free areas [5]. Misidentification of the pathogen could lead to drastic unnecessary measures, such as unjustified destruction of seeds or healthy biological material. On the other hand, a false-negative result could result in introduction of the pathogen to a new geographical area, increasing the risk of wheat blast outbreak.

However, intra-specific detection is challenging, since gene flow likely occurs regularly among lineages or subpopulations belonging to the same species. In the specific case of wheat blast, the detection method must be able to discriminate isolates responsible for wheat blast epidemics (belonging to the *Triticum* lineage) from isolates belonging to the other host-specific lineages of the species, but which may be capable of causing opportunistic infections on wheat plants. Host-specific lineages have identical morphology in pure culture, which does not enable the observer to differentiate them visually [20]. Pathotyping tests can be used for diagnosis but they are time-consuming and cannot identify opportunistic infections unambiguously. DNA-based detection tests are a good alternative because they allow detection at very precise taxonomic levels, provided, however, that they can identify a specific polymorphism in the targeted taxon. In the case of wheat blast, the identification of such polymorphisms is made difficult by the low genetic divergence and the gene flow between host-specific lineages [18].

Currently, several DNA-based diagnostic tests have been developed for the detection of wheat blast isolates [3,20,21]. All these tests are highly, but not perfectly, inclusive (i.e., detecting all wheat-blast strains) and/or specific (not detecting non-wheat blast isolates): none of them allows for optimal detection of the pathogen. Pieck et al. [3] and Yasuhara-Bell et al. [21] developed polymerase chain reaction (PCR), quantitative polymerase chain reaction (qPCR), and loop-mediated isothermal amplification (LAMP) diagnostic tests targeting the same genomic region, the MoT3 locus. This region was selected because it is highly conserved in wheat blast isolates and absent from most non-wheat blast isolates. However, some wheat blast isolates, such as the BR0032 isolate, do not include the MoT3 locus and remained undetectable using these tests. Thierry et al. [20] developed the C17 qPCR test targeting a different genomic region. This test allowed for the detection of all wheat blast isolates tested so far. However, some isolates that are not pathogenic on wheat were also detected with this test, leading to 4% false-positive results.

The objectives of this work were (i) to design primers targeting new genomic regions in order to identify polymorphisms fully specific to the *Triticum* lineage; (ii) to develop a toolkit of detection tests using multiple DNA amplification techniques (PCR, qPCR, and LAMP) to be suitable for any type of analysis and improving current wheat blast detection; and (iii) to verify the ability of these tests to detect the pathogen on artificially contaminated wheat grains.

## 2. Results

### 2.1. Primers Screening

#### 2.1.1. PCR Primers

Here, 49 designed primer pairs were first screened using conventional PCR on a small DNA panel composed of five wheat-borne isolates and ten non–wheat-borne isolates. Among them, 12 primer pairs displayed full inclusivity (i.e., positive result for all wheat-borne pathogenic isolates) and specificity (no detection of non-wheat borne pathogenic isolates; Figure S1). Four of these (C45, C74, C82, and C92) were selected for further analysis because they were designed on different regions/scaffolds of the reference genome BR0032 (scaffolds 15, 17, and 44).

#### 2.1.2. LAMP Primers

Five groups of primers were designed for a LAMP isothermal amplification. The inclusivity and specificity of the five groups of LAMP primers designed were tested on a small panel composed of three wheat-borne isolates and four non-wheat-borne isolates (Figure S2). None of the five groups of primers tested displayed full specificity and inclusivity. Two groups of primers did not amplify any of the DNA (groups 2 and 3) and two groups amplified all DNA extracts tested (groups 1 and 4). Group 5 was finally selected for further analysis because primers of this group made it possible to amplify every wheat-borne isolate in a very short time (around three minutes), even though full specificity of this primer group could not be achieved.

### 2.2. Primer Specificity and Inclusivity Assessment Using a Large DNA Panel

The inclusivity and specificity of the primers previously selected were assessed using a larger DNA panel of 113 or 185 strains (Table 1).

#### 2.2.1. PCR and qPCR Primers

The inclusivity and specificity of the C45, C74, C82, and C92 primer pairs were first assessed by conventional PCR (Table 1). The C45 primers displayed the highest inclusivity and specificity and were further assessed with additional DNAs (see Table 1) by conventional PCR, and by real-time PCR combing a hydrolysis probe (Table 1).

The C45 primers were designed in the scaffold 15 of the reference genome BR0032 and targeted a single SNP positioned at the 3’ end of the forward primer. PCR and qPCR tests using these primers and probe showed 97% inclusivity. All wheat-borne isolates but one (isolate AG0102) were amplified. To validate AG0102 host spectrum, a pathogenicity test was done by inoculating a spore suspension of AG0102 on the leaves of the susceptible wheat cultivar Thésée. No symptoms were observed, questioning the virulence of this isolate on wheat. Furthermore, no amplification of AG0102 DNA was observed using either the MoT3 test [3] or the C17 test [20] (Table 1).

C45 primers displayed full specificity by conventional PCR since no non-wheat-borne isolates of *P. oryzae* were amplified. However, late amplifications of DNA from non-wheat-borne isolates were observed when tested in real-time PCR. Additional qPCR repetitions with these DNA samples showed that these amplifications were not fully repeatable and only occurred erratically. Finally, qPCR testing using C45 primers allowed amplification of all wheat-borne isolates except AG0102 (Ct values between 23 and 28), but 10 out of 151 non–wheat-borne isolates were sometimes amplified with late Ct values (between 35 and 39). Specificity of the primers was also challenged with DNAs from other wheat infecting fungal species such as *Fusarium graminearum*, *Fusarium poae* or *Alternaria tenuissima*. None of these 19 DNAs were amplified using C45 primers either by conventional PCR or qPCR.

#### 2.2.2. LAMP Primers

LAMP primer group 5 successfully amplified DNA from all wheat-borne isolates, except the AG0102 isolate, in a very short time (between 3:37 and 4:10 min). However, as expected, full specificity could not be achieved. Six isolates (AG0067, Cd88215, CR0023, CR0057, JP0031, and JP0033) out of 151 (4%) were amplified with Ct values equivalent to the values observed with wheat-borne isolates, making them indistinguishable. Three of these six were also detected using the C17 qPCR test [20]. Late but non-repeatable amplifications could also be observed using the group 5 LAMP primers after 9 to 25 min of reaction (Table 1).

### 2.3. Assay Sensitivity

C45 PCR and qPCR primers: The sensitivity of PCR and qPCR primers was assessed using serial 10-fold dilutions of genomic DNA of three *P. oryzae* wheat-borne isolates (BR0031, BL0063, BL0023) and 10-fold dilutions of plasmidic constructions integrating the sequences targeted by the primers. C45 PCR and qPCR primers succeeded in detecting as low as 5 pg of DNA/reaction or 12 plasmidic copies/reaction for genomic DNA and plasmidic DNA, respectively. This limit of detection was validated using 16 replicates for each DNA concentration. PCR reaction efficiency was measured using the plasmidic dilution to 92% and the high R^2^ value (R^2^ = 0.9969) demonstrated good correlation between initial plasmidic DNA quantity and the Ct values.

Group 5 LAMP primers are as follows: the sensitivity of group 5 LAMP primers was assessed by serial 10-fold dilutions of initially diluted genomic samples of two *P. oryzae* wheat-borne isolates (BR0031 and BL0023). LAMP primers were able to amplify down to 5 pg of genomic DNA per reaction. This limit of detection was validated using five replicates.

### 2.4. Pathogen Detection in Contaminated Seeds

We assessed the ability of the PCR (C45 primers), qPCR (C45 primers + C45 probe), and LAMP (group No 5 primers) tests to detect the pathogen in a seed matrix. Wheat seeds were artificially contaminated with a spore suspension of the pathogen. Individual lots of 400 seeds were prepared containing 0, 1, 2, 5, 10, 15, or 50 artificially contaminated seeds mixed with non-contaminated seeds. Total DNA was extracted from each lot after blending. Before the blending step, some lots were incubated for 72 h in potato dextrose broth (PDB) media to test whether this enrichment process improved pathogen detection.

Without the incubation step, the conventional PCR succeeded in amplifying all nine replicates in seed lots containing 15 contaminated seeds or more. The real-time PCR test was less sensitive since it succeeded in amplifying all nine replicates only in seed lots containing 50 contaminated seeds or more. LAMP tests, on the other hand, failed to amplify DNA for any of the seed lots tested.

The incubation step greatly improved detection in all tests. After 72 h of incubation, the PCR test, as well as the qPCR test, were able to detect the pathogen for all replicates in seed lots containing only one contaminated seed. The LAMP primers allowed for the detection of the pathogen after an enrichment step, for all replicates, in lots containing two or more contaminated seeds in less than five min (Table 2).

Additionally, no non-specific late amplifications were obtained on seed lots highly contaminated by the BR0079 isolate (sampled on *Eleusine indica*). Seed lots containing 20 and 50 seeds contaminated with BR0079 spores did not yield a PCR or qPCR signal even after an incubation period of 72 h (Table 3).

## 3. Discussion

In this study, we designed primers targeting polymorphisms located in yet unexploited genomic regions in order to find a method with greater specificity for the *P. oryzae* Triticum lineage over the currently existing tests [3,20,21]. Screening these primers highlighted the C45 pair. The specificity of this pair is based on a single substitution located at the 720791 position of scaffold 15 in the BR0032 reference genome [22]. The isolates belonging to the *Triticum* lineage display a “C” at this position, while this nucleotide is substituted by a “G” in other lineages. In our conditions, this single substitution allowed the C45 pair to amplify the DNA of all the isolates sampled on wheat included in this study, with the exception of isolate AG0102 whose pathogenicity on wheat was questionable on the basis of our experiments. In addition, no amplification of DNA from *P. oryzae* isolates sampled from other Poaceae was found using conventional PCR, which demonstrates a higher level of specificity compared to the assays already described in the literature.

The use of these primers supplemented with a hydrolysis fluorescent probe enabled the development of a qPCR PCR test. Real-time PCR has two major advantages compared to conventional PCR: (i) it enables quantification of the pathogen in the samples tested and (ii) it reduces the turnaround time for analysis and offers reaction in close tubes, thus reducing the risk of self-contamination. The C45 qPCR test showed a similar level of inclusivity as conventional PCR (all isolates from wheat were amplified with the exception of AG0102). However, late but non-repeatable amplifications were observed for ten non-target isolates, with Ct higher than 35.5. The specificity of the targeted polymorphism is not called into question by these results. Importantly, the strong shift of Ct values between amplification of isolates sampled from wheat and the non-specific amplifications clearly demonstrates a difference in their nucleotide sequence. One explanation would be that, over time, a partial degradation of the primers could suppress the single nucleotide carrying the specificity and lead to these late amplifications during the amplification cycle. Late amplification obtained using the C45 qPCR test should be validated using other previously developed tests (MoT3: [3], C17: [20]) or by sequencing the locus. Clearly, a combination of several tests makes it possible to validate the results because different genomic regions are targeted by these tests, leading to different patterns of amplification (i.e., different false-positive or false-negative results).

The single substitution targeted by C45 did not allow the development of fully specific LAMP primers. The LAMP primers (group No 5) targeted another genomic region located at the 42,8000 position of the BR0032 scaffold 15. This test detected all isolates sampled on wheat, except isolate AG0102, in a very short time (<4 min). However, specificity assessed on 185 DNA samples from different *P. oryzae* isolates showed several non-specific amplifications. Six isolates (AG0067, Cd88215, CR0023, CR0057, JP0031, and JP0033) led to amplifications at similar times as the target *Triticum* isolates, between three and four min. As observed by Thierry et al. [20], some polymorphisms highly specific of the *Triticum* lineage identified on the scaffold 15 of the BR0032 isolate can still be shared with some isolates sampled on other grasses, like isolates CR0023, CR0057, and JP0033 [20]. Later, 22 other non-specific amplifications were observed after eight min of amplification. As for qPCR, late amplifications may be yielded by LAMP with DNA of these isolates due to rare unspecific bindings of the primers. In order to eliminate these late non-specific amplifications, the LAMP PCR run can be stopped at eight min without impacting the sensitivity of the test. However, the six non-specific amplifications observed between three and four min will not be eliminated by these modifications. Nonetheless, positive results can be confirmed with the C45 PCR or qPCR tests, which do not amplify DNA from these non-target isolates. The LAMP group 5 test could be used as a quick pre-screening, providing a result within eight min. If the result is positive on all replicates, then confirmation by PCR or qPCR should be done.

Knowing the sensitivity of a test is essential to validate the method. The limit of detection was very low for the three tests. The C45 PCR and qPCR tests as well as the LAMP group 5 test were able to detect as little as 5 pg of DNA per PCR reaction. The sensitivity of the C45 PCR and qPCR tests was also evaluated using plasmid DNA. The limit of detection obtained for both tests was 12 plasmid copies of the target DNA per PCR tube.

Detection of the pathogen on wheat grains is essential to validate the health status of seeds before trade and export or import. However, amplification inhibitors may be present in seeds and impact the performance of detection tests [23]. An enrichment phase in fungal biomass is often used to overcome these barriers [23,24]. In our study, only the C45 PCR and qPCR tests enabled repeatable amplification without an enrichment phase. Surprisingly, the C45 PCR test showed better sensitivity when used in a seed matrix (detection of 15 contaminated seeds per 400-seed lot for all replicates) than the qPCR test (detection of 50 contaminated seeds per 400-seed lot). The addition of a simple enrichment phase consisting of the incubation of grains for 72 h in a rich medium greatly improved the sensitivity of all the tests. Both the C45 PCR and qPCR tests allowed for systematic detection of the pathogen in a batch of 400 seeds containing only one artificially contaminated seed, which represents a 0.25% infection rate. The LAMP group 5 test allowed for systematic detection of the pathogen in lots containing two contaminated seeds after the enrichment phase.

In order to use the detection tests developed in this study to test for the presence of *P. oryzae* isolates belonging to the *Triticum* lineage on wheat seeds, lots of 400 seeds should be incubated for 72 h in a culture medium (PDB) prior to testing. DNA extracted from these grains will then serve as a template for detection tests. The detection method used may be selected depending on the financial, material, time and labor resources available. However, we recommend to first use fully inclusive detection tests, which do not generate false negatives on the isolates tested to date (C17, C45 PCR, C45 qPCR, and LAMP group 5 tests). The conventional C45 PCR test, however, is the only one that displayed perfect specificity for the isolates tested. Given the high genetic proximity of the host-specific lineages as well as the potential gene flow between these lineages, all positive results must be validated by the use of another test to avoid costly and unjustified quarantine measures, such as the complimentary approach proposed for the deadly *Fusarium oxysporum* f. sp. *cubense* race 4 on banana trees [25].

This study provides a new toolkit for the efficient detection of wheat-blast causal pathogen in seed, and therefore contributes to limit the spread of this destructive disease. The different types of techniques proposed may be of great help for laboratories in charge of phytosanitary controls and will allow to adapt to different levels of equipment.

## 4. Materials and Methods

### 4.1. Biological Materials

#### 4.1.1. *P. Oryzae* DNA

A total of 185 *P. oryzae* strains were screened to assess the specificity and the inclusivity of the diagnostic tests designed in this study. Among these *P. oryzae* isolates, 34 were sampled from field-infected wheat (*Triticum* sp.) and 151 were sampled from 28 different Poaceae genera. Isolates sampled from wheat are referred to as wheat-borne isolates in this article. Isolates were sampled in various countries, in order to cover the genetic diversity of the pathogen as much as possible and sent as DNA extracts to our lab. Included were 19 isolates belonging to nine other fungal species pathogenic on wheat (Table S1). All DNA concentrations were measured with a spectrophotometer (NanoDrop 2000; Thermo Fisher Scientific, Waltham, MA, USA) and adjusted to 0.5 ng/µL by dilution with a Tris EDTA 1X buffer. The amplifiability of all DNA was verified by the FungiQuant real-time PCR assay, which targeted a conserved 351 bp region of the fungal 18S rRNA gene [26].

#### 4.1.2. Positive Control Plasmids

Positive controls for the C45 PCR and qPCR detection tests were prepared by cloning the target amplicon in a plasmid. Plasmids are considered to be stable, homogeneous during pipetting, easily quantifiable, and producible in virtually unlimited quantities. The genomic region targeted by the C45 primer pair was amplified by PCR and the size of the amplicon checked on an electrophoresis gel. The amplicon was inserted into the pCR4-TOPO vector following the TOPO TA cloning kit protocol (Invitrogen) and used to transform TOPO10 chemically competent bacteria (*Escherichia coli*), according to the manufacturer’s instructions. After culturing the clones at 37 °C and selecting clones with fragment integration by qPCR, the plasmids were purified using a Nucleospin^®^ plasmid kit (Macherey-Nagel, Düren, Germany). The plasmid solution was used as a positive control for all PCR and qPCR reactions using the c45 couple.

### 4.2. Wheat Seed Inoculation and DNA Extraction

The wheat-borne isolate BL0092 was cultured on potato dextrose agar (PDA) medium at 23 °C, under day-night alternation (12 h/12 h). After seven days of culture, fungal spores were collected by adding 5 mL of sterile water to the Petri dish and scratching the mycelium to harvest the spores. The resulting suspension was filtered to remove mycelium fragments and retain only the conidia. Finally, the concentration of the suspension was calibrated using a haemocytometer at 200 spores/µL (supplemented by two drops of Tween 20)) and used to artificially inoculate wheat seeds of the susceptible wheat variety Filon. These seeds were previously disinfected by dipping for 10 min in a 1.5% active chlorine solution. The inoculation was carried out by depositing 10 μL of spore solution on individual grains and drying them overnight in a sterile atmosphere at room temperature. Multiple lots of 400 seeds in total but containing different proportions of contaminated seeds were created (0, 1, 2, 5, 10, 15, 20, or 50 artificially contaminated seeds per lot). Fungal enrichment was carried out by incubating the 400-grain lots in a sterile Petri dish containing 15 mL of potato dextrose broth (PDB) media for 72 h at room temperature (23 °C). Grinding of incubated or non-incubated seed lots was carried out with 30 mL of PDB using a Microtron™ MB 550 Laboratory Mixer (Kinematica™) until a homogenous milky mixture was obtained. For each mixture, three samples of 500 μL each were collected using a truncated 1 mL cone and used for DNA extraction using a Nucleospin^®^ Plant II extraction kit (Macherey-Nagel), following the manufacturer’s instructions. To serve as a negative control, the same spore inoculation protocol was performed with the BR0079 isolate sampled from *Eleusine indica*, and the DNA of two seed lots containing 20 and 50 inoculated seeds, respectively, were extracted after a fungal enrichment step.

### 4.3. Primer Design

PCR and qPCR: The primers and probes were designed to target *Triticum*-specific polymorphisms identified by comparison of 76 *P. oryzae* genomes, including 20 genomes assigned to the *Triticum* lineage. Here, we studied some of the polymorphisms highlighted but not exploited in the publication by Thierry et al. [20], as well as new polymorphisms further identified. Primers and probes for PCR and qPCR were designed using Geneious 11.1.2 software. The specific polymorphisms of the Triticum lineage were positioned at the 3′ end of the forward and reverse primers to maximize the specificity of the primers. The forward primer of the C45 pair was additionally purified by high performance liquid chromatography (HPLC) by the manufacturer to ensure the integrity of the primer, whose specificity is based on a single nucleotide located at the 3′ end of the forward primer. Primer sequences of the C45 test (PCR or qPCR) were C45-forward: 5′-TCTTTCACTCCTCCGAAAGAC-3′ and C45-reverse: 5′-GTATAGCTGGGTATCTTGGTAGAC-3′, whereas the combined hydrolysis probe in qPCR was C45-probe: 5′-FAM-TGCCCTCATCAAAACCTGCAGCCAT-BHQ1-3′.

LAMP: The four primers required for each LAMP reaction including the two external primers F3 and B3, and the two internal primers FIP (F1c + TTTT + F2) and BIP (B1c + TTTT + B2), were designed with the PrimerExplorer V5 online tool (https://primerexplorer.jp/e/). Sequences of the primers used for the group No.5 LAMP test were: No.5-F3: 5′-TGCGTGATCAACGAATGGC-3′; No.5-B3: 5′-CGGAAGCAAACTCTGCGATT-3′; No.5-FIP: 5′-GCAAGATGCCTACCGTGGGGTTTTCTGGGTTCCCCTCACCAT-3′; No.5-BIP: 5′-TCGTAAGGAGCATGAAGGGCTGTTTTGCGGACGGACATACGTAGT-3′.

All the primers used in this study were custom synthesized by Eurogentec. The list of all the candidate primers is available in Table S2.

### 4.4. Primer Screening

#### 4.4.1. PCR Primers

All primer pairs designed were screened using a small DNA panel from 15 isolates including five isolates sampled from *Triticum* (BR0086, BR0036, AG0103, BL0017 and BL0093), three from *Oryza* (CH1120, FR0013 and BR0019), four from *Lolium* (AG0064, CHW, PL2-1 and CR0057), one from *Eleusine* (IN0113), one from *Echinochloa* (CR0023), and one from *Eriochloa* (JP0033). The PCR reactions for the screening were carried out in qPCR conditions, without a hydrolysis probe, using a LightCycler^®^ 480 Probes Master (Roche), with the following reaction mixture: 1X Roche Kit qPCR premix, 0.3 μM of forward and reverse primers, 2 μL of template DNA (0.5 ng/μL), and ultrapure water for a final volume of 20 μL. The PCR reaction was carried out in a LightCycler^®^ 480 II thermal cycler (Roche). The PCR cycle was (i) an initial denaturation step at 95 °C for 10 min; (ii) 40 denaturation cycles at 95 °C for 15 s and hybridization-synthesis at 62 °C for 55 s; and (iii) a final elongation at 62 °C for 10 min.

#### 4.4.2. LAMP Primers

The small DNA panel used to screen LAMP primers was composed of seven DNA including the DNA of isolates sampled from *Triticum* (BR0036, BR0031 and BR0088), *Eriochloa* (JP0033), *Bromus* (AG0061), *Eleusine* (IN0113), and *Lolium* (CR0057). The reaction mix and amplification cycles used for this screening are described in the “PCR, qPCR, and LAMP amplifications” section below.

### 4.5. PCR, qPCR, and LAMP Amplifications

#### 4.5.1. Conventional PCR

After screening primer pairs using a small subset of DNAs, inclusivity and specificity of the remaining primers were tested on all 185 strains with the following conditions. The conventional PCR mix included 1X polymerase buffer, 5 mM MgCl2, 0.25 mM of each dNTP, 0.025 U/µL of HGS Diamond Taq^®^ DNA polymerase (Eurogentec, Seraing, Belgium), 0.3 µM of each forward and reverse primer, 2 µL of DNA matrix (0.5 ng/µL), and ultrapure water to reach a final 20 µL volume. The amplification cycle was 10 min of initial denaturation at 95 °C, followed by 40 cycles each composed of 30 s of denaturation at 95 °C, 30 s of hybridization at 65 °C and 45 s of extension at 72 °C, and a final extension at 65 °C for 10 min. PCR amplifications were visualized after a one-hour electrophoresis at 110 volts on a 1.5% agarose gel. Conventional PCR runs were carried out in a LightCycler^®^ 480 II thermal cycler (Roche).

#### 4.5.2. Real-Time PCR

The qPCR reactions were carried out on a RotorGene 4.4.1 (Qiagen, Hilden, Germany) using the following reaction mix: 1X No-ROX mastermix (Eurogentec), 0.3 µM of forward and reverse primers, 0.1 µM of hydrolysis fluorescent probe, 2 µL DNA matrix (0.5 ng/µL), and ultrapure water to reach a final volume of 20 µL. The qPCR amplification cycle included (i) a preliminary UNG glycosylase activation step at 50 °C for 2 min, (ii) an initial denaturation step at 95 °C for 10 min, and (iii) 40 cycles composed of 15 s of denaturation at 95 °C, and 55 s of hybridization-synthesis at 65 °C. The detection threshold was manually fixed at 0.02.

#### 4.5.3. LAMP

LAMP reactions were carried out using the following mix: 0.2 µM of each external primer (F3 et B3), 0.8 µM of each internal primer (FIP et BIP), 1X Isothermal Master Mix ISO-001 (OptiGene, Horsham, UK), 2 µL of DNA matrix (0.5 ng/µL), and ultrapure water to reach a final volume of 25 µL. LAMP being an isothermal amplification technique, amplifications were performed using a RotorGene 4.4.1thermocycler (Qiagen) whose temperature was set at 65 °C for 30 min. The detection threshold was manually fixed at 0.02.

## Figures and Tables

**Table 1:** Polymerase chain reaction (PCR), quantitative polymerase chain reaction (qPCR), and loop-mediated isothermal amplification (LAMP) primer screening using a large DNA panel. qPCR tests MoT3 [3] and C17 [20] are included for comparison. PCR results yielded positive (+) or negative (-) reaction, mean Ct values and standard deviation are indicated for qPCR and times for positive reaction are reported for LAMP. Isolates in red belong to other fungal species: *Microdochium nivale* (LSV M 641); *Fusarium tricinctum* (LSV M 723, LSV M 860); *Fusarium proliferatum* (LSV M 702, LSV M 706); *Fusarium poae* (LNPV 269, LSVM 861); *Fusarium graminearum* (LSV M 273, LSV M 811, LSV M 813); *Fusarium culmorum* (LSV M 662, LSV M 694, LSV M 697); *Fusarium avenaceum* (LSV M 642, LSV M 859, LSV M 863); *Alternaria tenuissima* (CBS918.96, CBS965.95) and *Alternaria alternata* (BRIP46550). Expected amplifications (positive results) are highlighted in green, late false positive amplification are highlighted in light red, whereas other false positive results as well as false negative results are highlighted in dark red. NT: not tested.

Isolates	Host of Origin	PCR C74	PCR C82	PCR C92	PCR C45	qPCR C45	LAMP n°5	qPCR MoT3	qPCR C17
AG0103	*Triticum aestivum*	+	+	+	+	26.13 ± 0.16	3:47 ± 0:00	+	+
BL0017	*Triticum aestivum*	+	+	+	+	25.46 ± 0.03	3:48 ± 0:00	+	+
BL0018	*Triticum aestivum*	+	+	+	+	25.29 ± 0.05	3:51 ± 0:03	+	+
BL0020	*Triticum aestivum*	+	+	+	+	25.04 ± 0.05	3:53 ± 0:00	+	+
BL0023	*Triticum aestivum*	+	+	+	+	25.33 ± 0.10	3:54 ± 0:01	+	+
BL0028	*Triticum aestivum*	+	+	+	+	25.19 ± 0.20	3:53 ± 0:00	+	+
BL0037	*Triticum aestivum*	+	+	+	+	24.64 ± 0.06	3:55 ± 0:01	+	+
BL0044	*Triticum aestivum*	+	+	+	+	24.46 ± 0.19	3:55 ± 0:01	+	+
BL0046	*Triticum aestivum*	+	+	+	+	25.22 ± 0.20	3:52 ± 0:00	+	+
BL0063	*Triticum aestivum*	+	+	+	+	25.01 ± 0.05	3:54 ± 0:01	+	+
BL0092	*Triticum* sp.	+	+	+	+	23.75 ± 0.18	3:56 ± 0:01	+	+
BL0093	*Triticum* sp.	+	+	+	+	24.45 ± 0.20	3:52 ± 0:02	+	+
BR0031	*Triticum* sp.	+	-	+	+	24.67 ± 0.15	3:52 ± 0:02	+	+
BR0032	*Triticum* sp.	+	+	+	+	23.22 ± 0.30	3:57 ± 0:00	-	+
BR0034	*Triticum* sp.	-	+	+	+	24.43 ± 0.11	3:53 ± 0:01	+	+
BR0036	*Triticum* sp.	+	+	+	+	25.30 ± 0.13	3:48 ± 0:01	+	+
BR0039	*Triticum* sp.	-	+	+	+	24.61 ± 0.05	3:50 ± 0:02	+	+
BR0040	*Triticum* sp.	+	+	+	+	25.49 ± 0.10	3:51 ± 0:05	+	+
BR0041	*Triticum* sp.	-	+	+	+	26.30 ± 0.05	3:47 ± 0:03	+	+
BR0043	*Triticum* sp.	+	+	+	+	24.60 ± 0.13	3:55 ± 0:03	-	+
BR0045	*Triticum* sp.	+	+	+	+	27.79 ± 0.12	3:40 ± 0:03	+	+
BR0047	*Triticum* sp.	-	+	+	+	25.51 ± 0.06	3:53 ± 0:01	+	+
BR0080	*Triticum* sp.	+	+	+	+	25.09 ± 0.06	3:53 ± 0:00	+	+
BR0086	*Triticum* sp.	+	+	+	+	24.84 ± 0.21	3:51 ± 0:03	+	+
BR0087	*Triticum* sp.	+	+	+	+	25.76 ± 0.29	3:51 ± 0:03	+	+
BR0088	*Triticum* sp.	+	+	+	+	24.58 ± 0.05	3:54 ± 0:01	+	+
BR0123	*Triticum aestivum*	+	+	+	+	24.61 ± 0.07	3:55 ± 0:01	+	+
BTGP16	*Triticum* sp.	+	+	+	+	25.35 ± 0.18	3:70 ± 0:44	+	+
BTJP4-1	*Triticum* sp.	+	+	+	+	25.33 ± 0.07	3:38 ± 0:00	+	+
BTMP13-1	*Triticum* sp.	+	+	+	+	25.52 ± 0.25	3:37 ± 0:00	+	+
AG0102	*Triticum aestivum*	NT	NT	NT	-	-	-	NT	NT
BL0042	*Triticum aestivum*	NT	NT	NT	+	26.24 ± 0.09	3:48 ± 0:09	NT	NT
BL0066	*Triticum aestivum*	NT	NT	NT	+	25.55 ± 0.23	3:50 ± 0:01	NT	NT
BL0074	*Triticum aestivum*	NT	NT	NT	+	26.31 ± 0.08	3:41 ± 0:01	NT	NT
AG0054	*Bromus* sp.	+	+	+	-	-	10:39	-	-
AG0055	*Bromus* sp.	+	-	-	-	-	-	+	-
AG0061	*Bromus unioloides*	-	+	+	-	-	-	-	-
AG0062	*Lolium* sp.	-	-	-	-	-	-	-	-
AG0063	*Lolium* sp.	-	-	-	-	-	10:82 ± 0:52	-	-
AG0064	*Lolium* sp.	-	-	-	-	-	-	-	-
AG0065	*Stenotaphrum* sp.	-	-	-	-	-	-	-	-
AG0132	*Oryza sativa*	-	-	-	-	-	-	-	-
BF0017	*Pennisetum typhoides*	-	-	-	-	-	-	-	-
BR0019	*Oryza sativa*	-	-	-	-	-	-	-	-
BR0029	*Digitaria sanguinalis*	-	-	-	-	-	-	-	-
BR0030	*Cenchrus echinatus*	-	-	-	-	-	-	-	-
BR0062	*Eleusine indica*	-	-	-	-	-	-	-	-
BR0070	*Eragrostis* sp.	-	-	-	-	-	-	-	-
Br58	*Avena* sp.	-	-	-	-	37.04 ± 2.02	-	-	-
CD0143	*Digitaria exilis*	-	-	-	-	-	-	-	-
Cd88215	*Echinochloa colona*	-	-	-	-	-	3:65 ± 0:12	-	-
CH0333	*Oryza sativa*	-	-	-	-	-	-	-	-
CH1120	*Oryza sativa*	-	-	-	-	-	-	-	-
LcA8401	*Leptochloa chinensis*	-	-	-	-	37.49 ± 1.68	-	NT	NT
CHRF	*Lolium* sp.	-	-	-	-	-	9:66	-	-
CHW	*Lolium* sp.	-	-	-	-	-	-	-	-
CR0021	*Panicum miliaceum*	-	-	-	-	-	-	-	-
CR0023	*Echinochloa crus-galli*	-	-	-	-	-	3:52 ± 0:04	-	+
CR0026	*Lolium* sp.	-	-	-	-	-	-	-	-
CR0029	*Festuca elalior*	-	-	-	-	-	-	-	-
CR0030	*Setaria viridis*	-	-	-	-	-	-	-	-
CR0031	*Setaria italica*	-	-	-	-	-	-	-	-
CR0057	*Lolium* sp.	-	-	-	-	-	3:59 ± 0:02	-	+
EG0028	*Cyperus rotundus*	-	-	-	-	-	-	-	-
FH	*Lolium* sp.	-	-	-	-	-	-	-	-
FR0013	*Oryza* sp.	-	-	-	-	-	-	-	-
FR1069	*Lolium* sp.	-	-	-	-	-	-	-	-
GG11	*Lolium* sp.	-	-	-	-	-	-	-	-
GN0001	*Zea mays*	-	-	-	-	-	-	-	-
GR0001	*Ctenanthe oppenheimiana*	-	-	-	-	-	-	-	-
GY0011	*Oryza sativa*	-	-	-	-	-	-	-	-
HO	*Lolium* sp.	-	-	-	-	-	-	-	-
IN0003	*Panicum repens*	-	-	-	-	-	-	-	-
IN0005	*Panicum maximun*	+	-	-	-	-	-	-	-
IN0022	*Setaria* sp.	-	-	-	-	-	13:10	-	-
IN0023	*Setaria* sp.	-	-	-	-	-	-	-	-
IN0082	*Oryza sativa*	-	-	-	-	-	-	-	-
IN0108	*Setaria* sp.	-	-	-	-	-	-	-	-
IN0113	*Eleusine* sp.	-	-	-	-	-	-	-	-
IN0115	*Oryza sativa*	-	-	-	-	-	-	-	-
IR0013	*Zea mays*	-	-	-	-	-	-	-	-
IR0015	*Zea mays*	-	-	-	-	-	-	-	-
IR0095	*Zea mays*	-	-	-	-	-	-	-	-
IR0102	*Echinochloa* sp.	-	-	-	-	-	-	-	-
IS0001	*Cyperus rotundus*	-	-	-	-	-	-	-	-
JP0028	*Eragrostis curvula*	-	-	-	-	-	23:53	-	-
JP0030	*Panicum bisulcatum*	-	-	-	-	-	17:69	-	-
JP0031	*Panicum coloratum*	-	-	-	-	-	3:29 ± 0:50	-	-
JP0033	*Eriochloa villosa*	-	-	-	-	-	3:68 ± 0:04	-	+
JP0047	*Hordeum vulgare*	-	-	-	-	-	-	-	-
JP0048	*Hordeum vulgare*	-	-	-	-	-	-	-	-
KN0001	*Hordeum vulgare*	-	-	-	-	-	-	-	-
KN0006	*Hordeum vulgare*	-	-	-	-	-	-	-	-
Lc8401	*Leptochloa chimensis*	-	-	-	-	-	-	-	-
LpKY97	*Lolium* sp.	-	-	-	-	-	-	-	-
ML0031	*Pennisetum* sp.	-	-	-	-	-	-	-	-
Pd88413	*Paspalum distichum*	-	-	-	-	-	-	-	-
Pg1054	*Stenotaphrum secundatum*	-	-	-	-	-	-	-	-
Pg1213-22	*Festuca* sp.	-	-	-	-	-	-	-	-
PH0052	*Cyperus rotundus*	-	-	-	-	-	-	-	-
PH0053	*Cyperus rotundus*	-	-	-	-	-	-	-	-
PH0062	*Paspalum distichum*	-	-	-	-	-	-	-	-
PH0075	*Brachiaria mutica*	-	-	-	-	-	-	-	-
PH0078	*Echinochloa* sp.	-	-	-	-	-	-	-	-
PH0097	*Paspalum paspaloides*	-	-	-	-	-	-	-	-
PL 2-1	*Lolium* sp.	-	-	-	-	-	-	-	-
PL 3-1	*Lolium* sp.	-	-	-	-	-	-	-	-
PR0069	*Stenotaphrum secundatum*	-	-	-	-	-	-	-	-
PrA8202	*Panicum repens*	-	-	-	-	-	-	-	-
RW0043	*Eleusine coracana*	-	-	-	-	-	12:85	-	-
TFO5-1	*Festuca* sp.	-	-	-	-	-	-	-	-
US0064	*Setaria* sp.	-	-	-	-	-	-	-	-
US0066	*Cenchrus ciliaris*	-	-	-	-	-	-	-	-
US0077	*Lolium perenne*	-	-	-	-	-	-	-	-
US0078	*Lolium perenne*	-	-	-	-	-	-	-	-
US0084	*Stenotaphrum secundatum*	-	-	-	-	-	-	-	-
VT0032	*Leersia hexandra*	-	-	-	-	-	-	-	-
AG0049	*Echinochloa* sp	NT	NT	NT	-	-	-	NT	NT
AG0050	*Echinochloa* sp	NT	NT	NT	-	-	-	NT	NT
AG0051	*Echinochloa* sp	NT	NT	NT	-	-	-	NT	NT
AG0058	*Eleusine indica*	NT	NT	NT	-	-	-	NT	NT
AG0059	*Eleusine indica*	NT	NT	NT	-	-	-	NT	NT
AG0067	*Phalaris canariense*	NT	NT	NT	-	-	3:68 ± 0:03	NT	NT
AU0002	*Oryza rufipogon*	NT	NT	NT	-	37.57 ± 1.30	8:51 ± 0:29	NT	NT
BF0026	*Eleusine indica*	NT	NT	NT	-	36.07 ± 1.68	-	NT	NT
BF0080	*Oryza longistaminata*	NT	NT	NT	-	-	-	NT	NT
BF0083	*Oryza longistaminata*	NT	NT	NT	-	-	-	NT	NT
BF0093	*Oryza longistaminata*	NT	NT	NT	-	-	-	NT	NT
BF0181	*Oryza longistaminata*	NT	NT	NT	-	-	-	NT	NT
BG0007	*Leersia hexandra*	NT	NT	NT	-	-	-	NT	NT
BG0023	*Leersia hexandra*	NT	NT	NT	-	-	-	NT	NT
BG0024	*Leersia hexandra*	NT	NT	NT	-	-	-	NT	NT
BR0066	*Eleusine indica*	NT	NT	NT	-	-	10:12 ± 0:88	NT	NT
BR0071	*Echinochloa* sp	NT	NT	NT	-	38.56 ± 0.54	-	NT	NT
BR0079	*Eleusine indica*	NT	NT	NT	-	38.48 ± 1.09	-	NT	NT
BR0093	*Echinochloa colona*	NT	NT	NT	-	-	-	NT	NT
CD0060	*Oryza glaberrima*	NT	NT	NT	-	-	15:62	NT	NT
CD0157	*Eleusine indica*	NT	NT	NT	-	37.80 ± 1.64	-	NT	NT
CD0258	*Leersia hexandra*	NT	NT	NT	-	-	-	NT	NT
CH0321	*Oryza sativa*	NT	NT	NT	-	-	-	NT	NT
CH0328	*Oryza sativa*	NT	NT	NT	-	-	-	NT	NT
CH0331	*Oryza sativa*	NT	NT	NT	-	-	-	NT	NT
CH0338	*Oryza sativa*	NT	NT	NT	-	-	-	NT	NT
CH0341	*Oryza sativa*	NT	NT	NT	-	-	-	NT	NT
CL0013	*Echinochloa colona*	NT	NT	NT	-	-	8:67 ± 0:04	NT	NT
CL0045	*Rottboellia exalta*	NT	NT	NT	-	-	11:45 ± 1:15	NT	NT
CL0089	*Oryza rufipogon*	NT	NT	NT	-	35.50 ± 2.39	-	NT	NT
CR0058	*Setaria viridis*	NT	NT	NT	-	-	-	NT	NT
CR0060	*Eleusine indica*	NT	NT	NT	-	-	11:96 ± 0:75	NT	NT
EG0025	*Echinochloa colona*	NT	NT	NT	-	-	-	NT	NT
FR1067	*Lolium perenne*	NT	NT	NT	-	-	21:75	NT	NT
GD0001	*Eleusine indica*	NT	NT	NT	-	-	-	NT	NT
IN0004	*Panicum repens*	NT	NT	NT	-	-	-	NT	NT
IN0030	*Echinochloa frumentaceum*	NT	NT	NT	-	-	-	NT	NT
JP0020	*Eleusine indica*	NT	NT	NT	-	36.82 ± 2.22	-	NT	NT
JP0035	*Pennisetum clandesti*	NT	NT	NT	-	-	-	NT	NT
JP0036	*Leersia hexandra*	NT	NT	NT	-	-	-	NT	NT
JP0039	*Anthoxanthum odoratum*	NT	NT	NT	-	-	10:23	NT	NT
JP0040	*Phalaris arundinacea*	NT	NT	NT	-	-	-	NT	NT
JP0098	*Setaria faberii*	NT	NT	NT	-	-	21:96	NT	NT
JP0102	*Setaria faberii*	NT	NT	NT	-	-	-	NT	NT
MD0112	*Eleusine indica*	NT	NT	NT	-	-	11:91 ± 2:40	NT	NT
MD0153	*Eleusine indica*	NT	NT	NT	-	-	-	NT	NT
ML0070	*Oryza longistaminata*	NT	NT	NT	-	-	-	NT	NT
ML0074	*Oryza longistaminata*	NT	NT	NT	-	-	-	NT	NT
NP0060	*Eleusine coracana*	NT	NT	NT	-	-	-	NT	NT
NR0041	*Oryza longistaminata*	NT	NT	NT	-	-	-	NT	NT
NR0049	*Leersia hexandra*	NT	NT	NT	-	-	-	NT	NT
PH0035	*Brachiaria mutica*	NT	NT	NT	-	-	18:42 ± 9:59	NT	NT
PH0045	*Brachiaria mutica*	NT	NT	NT	-	-	-	NT	NT
PH0046	*Brachiaria distachya*	NT	NT	NT	-	-	-	NT	NT
PH0056	*Echinochloa ciliaris*	NT	NT	NT	-	-	11:81 ± 0:67	NT	NT
PH0057	*Eleusine indica*	NT	NT	NT	-	-	-	NT	NT
PH0077	*Echinochloa colona*	NT	NT	NT	-	-	-	NT	NT
PH0079	*Panicum repens*	NT	NT	NT	-	-	-	NT	NT
PH0080	*Panicum repens*	NT	NT	NT	-	-	-	NT	NT
PH0081	*Paspalum paspaloides*	NT	NT	NT	-	-	12:19 ± 2:68	NT	NT
PR0083	*Stenotaphrum secondatum*	NT	NT	NT	-	-	-	NT	NT
RN0001	*Zingiber officinale*	NT	NT	NT	-	-	-	NT	NT
RW0018	*Eleusine coracana*	NT	NT	NT	-	37.29 ± 2.65	13:56	NT	NT
RW0022	*Eleusine indica*	NT	NT	NT	-	-	-	NT	NT
RW0031	*Eleusine coracana*	NT	NT	NT	-	-	-	NT	NT
RW0036	*Eleusine coracana*	NT	NT	NT	-	-	-	NT	NT
RW0038	*Eleusine coracana*	NT	NT	NT	-	-	-	NT	NT
RW0041	*Eleusine coracana*	NT	NT	NT	-	-	25:09	NT	NT
LSV M 641	*Triticum* sp.	NT	NT	NT	-	-	NT	NT	NT
LSV M 723	*Triticum* sp.	NT	NT	NT	-	-	NT	NT	NT
LSV M 860	*Triticum* sp.	NT	NT	NT	-	-	NT	NT	NT
LSV M 702	*Zea mays*	NT	NT	NT	-	-	NT	NT	NT
LSV M 706	*Zea mays*	NT	NT	NT	-	-	NT	NT	NT
LNPV 269	*Triticum* sp.	NT	NT	NT	-	-	NT	NT	NT
LSVM 861	*Triticum* sp.	NT	NT	NT	-	-	NT	NT	NT
LSV M 273	*Triticum* sp.	NT	NT	NT	-	-	NT	NT	NT
LSV M 811	*Triticum* sp.	NT	NT	NT	-	-	NT	NT	NT
LSV M 813	*Triticum* sp.	NT	NT	NT	-	-	NT	NT	NT
LSV M 662	*Triticum* sp.	NT	NT	NT	-	-	NT	NT	NT
LSV M 694	*Triticum* sp.	NT	NT	NT	-	-	NT	NT	NT
LSV M 697	*Triticum* sp.	NT	NT	NT	-	-	NT	NT	NT
LSV M 642	*Triticum* sp.	NT	NT	NT	-	-	NT	NT	NT
LSV M 859	*Triticum* sp.	NT	NT	NT	-	-	NT	NT	NT
LSV M 863	*Triticum* sp.	NT	NT	NT	-	-	NT	NT	NT
CBS918.96	*Dianthus chioensis*	NT	NT	NT	-	-	NT	NT	NT
CBS965.95	*Triticum* sp.	NT	NT	NT	-	-	NT	NT	NT
BRIP46550	*Malus* sp.	NT	NT	NT	-	-	NT	NT	NT

**Table 2:** Sensitivity of the PCR (C45 primers), qPCR (C45 primers), and LAMP (group 5 primers) tests in seed matrix contaminated with a *Triticum* lineage *P. oryzae* strain (BL0092).

BL0092	Number of Contaminated Grains Per Lot	C45 PCR	C45 qPCR		Group 5 LAMP	
		Positive Replicates/Total Replicates	Positive Replicates/Total Replicates	Mean Ct ± SD	Positive Replicates/Total Replicates	min ± SD
	0 grain	0/9	0/9	>40	0/4	-
0 h	1 grain	1/9	3/9	37.21 ± 0.74	0/4	-
	2 grains	2/9	1/9	37.31	0/4	-
	5 grains	3/9	4/9	37.66 ± 1.45	0/4	-
	10 grains	6/9	7/9	37.16 ± 0.88	0/4	-
	15 grains	9/9	6/9	36.74 ± 1.17	1/4	13:74
	50 grains	9/9	9/9	35.36 ± 0.60	NT	NT
	0 grain	0/9	0/9	>40	0/4	-
72 h	1 grain	9/9	9/9	33.83 ± 0.61	2/4	12:97 ± 10:80
	2 grains	9/9	9/9	30.97 ± 0.41	4/4	4:66 ± 0:55
	5 grains	9/9	9/9	29.00 ± 0.32	4/4	4:24 ± 0:09
	10 grains	9/9	9/9	29.32 ± 0.25	4/4	4:22 ± 0:18
	15 grains	9/9	9/9	28.56 ± 0.25	4/4	4:13 ± 0:24
	50 grains	9/9	9/9	26.47 ± 0.32	NT	NT

**Table 3:** Sensitivity of the PCR (C45 primers), qPCR (C45 primers), and LAMP (group 5 primers) tests in seed matrix contaminated with a non-target *P. oryzae* strain from *E. indica* (BR0079).

BR0079	Number of Contaminated Grains Per Lot	PCR	qPCR	Group 5 LAMP
		Positive Replicates/Total Replicates	Positive Replicates/Total Replicates	
**72 h**	20 grains	0/9	0/9	NT
	50 grains	0/9	0/9	NT

## Supplementary Materials

The following are available online at https://www.mdpi.com/2223-7747/9/2/277/s1, Figure S1: PCR primer screening using a small panel. Green: specific amplification; red: non-specific amplification; white: no amplification; Figure S2: LAMP primer screening using a small panel. Green: specific amplification; red: non-specific amplification (min); Table S1: List of DNA used in this study; Table S2: List of candidate primers and probes used in this study.

## References

[1] Urashima A.S., Igarashi S., Kato H. (1993). Host range, mating type, and fertility of *Pyricularia grisea* from wheat in Brazil. Plant Dis..

[2] Islam M.T., Kim K.-H., Choi J. (2019). Wheat blast in Bangladesh: The current situation and future Impacts. Plant Pathol. J..

[3] Pieck M.L., Ruck A., Farman M.L., Peterson G.L., Stack J.P., Valent B., Pedley K.F. (2017). Genomics-based marker discovery and diagnostic assay development for wheat blast. Plant Dis..

[4] Urashima A.S., Lavorent N.A., Goulart A.C.P., Mehta Y.R. (2004). Resistance spectra of wheat cultivars and virulence diversity of *Magnaporthe grisea* isolates in Brazil. Fitopatol. Bras..

[5] Cruz C.D., Valent B. (2017). Wheat blast disease: Danger on the move. Trop. Plant Pathol..

[6] FAOSTAT. http://www.fao.org/faostat/fr/#home.

[7] Igarashi S., Utiamada C.M., Kasuma A.H., Lopez R.S. (1986). *Pyricularia* em trigo. 1. Ocorrência de *Pyricularia* sp. no estado do Paraná. Fitopatol. Bras..

[8] Anjos J., Da Silva D.B., D’Avila Charchar M.J., Rodrigues G.C. (1996). Occurrence of blast fungus (*Pyricularia grisea*) on wheat and rye in the savanna region of central Brazil. Pesq. Agropec. Bras..

[9] Goulart A. (1992). Incidência da brusone (*Pyricularia oryzae*) em diferentes cultivares de trigo (*Triticum aestivum*) em condições de campo. Fitopatol. Bras..

[10] Goulart A.C.P., Sousa P.G., Urashima A.S. (2007). Damages in wheat caused by infection of *Pyricularia grisea*. Summa Phytopathol..

[11] Piccinini E.C., Fernandes J.M.C. (1990). Ocorrencia da brusone (*Pyricularia oryzae*) em lavouras comerciais de trigo (*Triticum aestivum*) no estado do Rio Grande do Sul, Brazil. Fitopatol. Bras..

[12] Islam M.T., Croll D., Gladieux P., Soanes D.M., Persoons A., Bhattacharjee P., Hossain M.S., Gupta D.R., Rahman M.M., Mahboob M.G. (2016). Emergence of wheat blast in Bangladesh was caused by a South American lineage of *Magnaporthe oryzae*. BMC Biol..

[13] Malaker P.K., Barma N.C.D., Tiwari T.P., Collis W.J., Duveiller E., Singh P.K., Joshi A.K., Singh R.P., Braun H.-J., Peterson G.L. (2016). First report of wheat blast caused by *Magnaporthe oryzae* pathotype triticum in Bangladesh. Plant Dis..

[14] Urashima A.S., Leite S.F., Galbieri R. (2007). Eficiência da disseminação aérea em *Pyricularia grisea*. Summa Phytopathol..

[15] Gomes D.P., Rocha V.S., Rocha J.R.D.A.S.D.C., Pereira O.L., Souza M.A.D. (2018). Potential of transmission of *Pyricularia graminis-tritici* from plant to seed and from seed to seedling in wheat genotypes with different degrees of blast resistance. J. Seed Sci..

[16] Klaubauf S., Tharreau D., Fournier E., Groenewald J.Z., Crous P.W., de Vries R.P., Lebrun M.-H. (2014). Resolving the polyphyletic nature of *Pyricularia* (Pyriculariaceae). Stud. Mycol..

[17] Ou S.H. (1985). Rice Diseases.

[18] Gladieux P., Condon B., Ravel S., Soanes D., Maciel J.L.N., Nhani A., Chen L., Terauchi R., Lebrun M.-H., Tharreau D. (2018). Gene flow between divergent cereal-and grass-specific lineages of the rice blast fungus *Magnaporthe oryzae*. MBio.

[19] Farman M., Peterson G., Chen L., Starnes J., Valent B., Bachi P., Murdock L., Hershman D., Pedley K., Fernandes J.M. (2016). The *Lolium* pathotype of *Magnaporthe oryzae* recovered from a single blasted wheat plant in the United States. Plant Dis..

[20] Thierry M., Gladieux P., Fournier E., Tharreau D., Ioos R. (2019). A genomic approach to develop a new qPCR test enabling detection of the *Pyricularia oryzae* lineage causing wheat blast. Plant Dis..

[21] Yasuhara-Bell J., Pedley K.F., Farman M., Valent B., Stack J.P. (2018). Specific detection of the wheat blast pathogen (*Magnaporthe oryzae Triticum*) by loop-mediated isothermal amplification. Plant Dis..

[22] Chiapello H., Mallet L., Guérin C., Aguileta G., Amselem J., Kroj T., Ortega-Abboud E., Lebrun M.H., Henrissat B., Gendrault A. (2015). Deciphering genome content and evolutionary relationships of isolates from the fungus *Magnaporthe oryzae* attacking different host plants. Genome Biol. Evol..

[23] Mancini V., Murolo S., Romanazzi G. (2016). Diagnostic methods for detecting fungal pathogens on vegetable seeds. Plant Pathol..

[24] Ioos R., Fourrier C., Iancu G., Gordon T.R. (2009). Sensitive detection of *Fusarium circinatum* in pine seed by combining an enrichment procedure with a real-time polymerase chain reaction using dual-labeled probe chemistry. Phytopathology.

[25] Magdama F., Monserrate-Maggi L., Serrano L., Sosa D., Geiser D.M., Jiménez-Gasco M.D.M. (2019). Comparative analysis uncovers the limitations of current molecular detection methods for *Fusarium oxysporum* f. sp. cubense race 4 strains. PLoS ONE.

[26] Liu C.M., Kachur S., Dwan M.G., Abraham A.G., Aziz M., Hsueh P.-R., Huang Y.-T., Busch J.D., Lamit L.J., Gehring C.A. (2012). FungiQuant: A broad-coverage fungal quantitative real-time PCR assay. BMC Microbiol..

